# Dissociation of prepotent response inhibition and interference control in problematic internet use: evidence from the Go/No-Go and Flanker tasks

**DOI:** 10.1186/s40359-024-01698-6

**Published:** 2024-04-18

**Authors:** Shao-Shuai Zhang, Yu-qing Zhong, Xu Li, Ming Peng

**Affiliations:** 1 Key Laboratory of Adolescent Cyberpsychology and Behavior Central China Normal University (CCNU), Ministry of Education, Wuhan, China; 2 Hubei Human development and mental health key Laboratory (Central China Normal University), School of Psychology, Central China Normal University, No. 382, XiongChu Road, Hongshan District, 430079 Wuhan, Hubei Province China

**Keywords:** Problematic internet use, Inhibitory control, Prepotent response inhibition, Interference control, Distractor resistance

## Abstract

**Background:**

Problematic Internet Use (PIU), characterized by failures to control the overuse of internet, is associated with a range of functional impairments. However, there is limited research on the specific impact of PIU on inhibitory control functions, particularly in terms of differentiating between prepotent response inhibition and interference control. Therefore, the main objective of this study is to investigate these two components of inhibitory control in individuals with PIU.

**Methods:**

Thirty participants who met the PIU criteria and 30 control participants were included in the present study. All participants completed the Go/No-Go and Flanker tasks, in which internet-related images and words were used as task stimuli.

**Results:**

In the Go/No-Go task, all participants exhibited poorer performance in inhibiting internet-related stimuli compared to internet-unrelated stimuli, during the No-Go trials. In the Flanker task, results revealed a three-way interaction of Group, Stimulus type and Congruency. Specifically, in the incongruent condition, participants with PIU exhibited slower responses for internet-unrelated targets compared to internet-related targets, whereas no similar effect was observed among individuals with low internet use.

**Conclusions:**

The findings suggest that difficulties in controlling the interference effect of internet-related information represent a key dysfunction in inhibitory control of PIU.

## Background

The global internet user population has reached a staggering 5.19 billion individuals, representing approximately 64.5% of the total global population (Datareportal; https://datareportal.com/global-digitaloverview). While this digital expansion has facilitated vast opportunities such as information exchange and social connection, it has also been paralleled with issues related to internet overuse. Problematic Internet Use (PIU) is characterized by excessive and compulsive engagement with the internet, leading individuals to exhibit obsessive preoccupation, difficulty in reducing or discontinuing online activities, and a tendency to isolate themselves from real-life interactions [1]. Contrasting with Internet Gaming Disorder (IGD), which is specifically focused on excessive gaming online, PIU provides a broader perspective on internet-related disorders [2]. Importantly, PIU has been recognized as a prominent contributing factor to a range of functional impairments and mental health concerns. For example, PIU has been associated with heightened sleep disturbances, elevated levels of depressive and anxiety symptoms, and increased feelings of loneliness [3], all of which further exert detrimental effects on individuals’ academic and work performance, as well as their overall daily functioning.

Inhibitory control, a subcomponent of executive functions, encompasses the ability to suppress habitual, dominant, and prepotent responses that are deemed inappropriate within a given context. It also involves the capacity to resist interference caused by distractors [4]. Deficits in inhibitory control are proposed to underpin cognitive dysfunctions in many mental disorders such as attention deficit hyperactivity disorder, obsessive compulsive disorder and substance abuse. Importantly, previous research has established a connection between PIU and prominent inhibitory control failures, for example, individuals with PIU experience difficulties in controlling their impulsivity to use the internet, struggle to resist cravings for prolonged internet use, and display withdrawal-like symptoms when unable to access the internet. Empirical studies have reported positive correlations between the self-report severity of impulsivity symptoms and PIU [5, 6]. Additionally, it has been proposed that the impulsive symptoms observed in individuals with PIU may be indicative of an underlying deficiency in inhibitory control [7], implicating that disrupted inhibitory control serving as a fundamental cognitive vulnerability factor for PIU.

It is noteworthy that inhibitory control, although often treated as a unitary construct, is increasingly recognized as a multifaceted phenomenon according to research from cognitive and behavioral neuroscience, and the two most commonly recognized components are prepotent response inhibition and interference control [8, 9]. Prepotent response inhibition refers to the suppression of dominant responses, involving the inhibition of prepotent but inappropriate responses. On the other hand, interference control refers to the ability to suppress interference from goal-irrelevant information. This distinction is supported by numerous studies in both healthy populations and various clinical samples, such as schizophrenia, autism spectrum disorder, depression, and obsessive-compulsive disorder. The Go/No-Go task and the stop-signal task are consistently classified as measures of prepotent response inhibition [10], in which participants are required to cancel or withdraw ongoing responses, while the Flanker task, the Simon task are widely used as indices of interference control, in which participants are instructed to exert inhibitory control by suppressing interference from irrelevant Flankers. In addition, the Stroop task, which involves conflict monitoring and interference suppression, has also been used to assess interference control, despite the ongoing controversies surrounding the inhibitory mechanisms that underpin this task and its limited convergent validity when compared to other measures [8, 11].

The majority of previous research has explored the relationship between PIU and prepotent response inhibition [10]. For example, participants with PIU show poor performance than controls in the Go/No-Go task [12, 13] and have lower proportion of successful stop in the stop-signal task [14]. However, some other studies have reported no differences between participants with and without PIU on response inhibition, either in the Go/No-Go or in the stop-signal task [2, 15]. The inconsistency might be related to task materials. For example, in the study by Nie et al. [16], it was found participants with PIU made more errors in stop trials for the internet-related words compared to internet-unrelated words. In the study by Chamberlain et al. [15], arrows, which were internet-unrelated, were used in the stop-signal task, and no differences on stop-signal reaction times were found between participants with PIU and controls. Studies using digit or letter as task materials in the Go/No-Go task also found no differences regarding accuracy of No-Go trials or response times of Go trials between participants with PIU and controls [2, 17]. These results highlight that PIU might be related to specific deficits in inhibiting internet-related information rather than general deficits in prepotent response inhibition.

With regard to interference control function in PIU, very few studies have been conducted. In a study using the Flanker task, it is found that individuals with PIU performed worse than healthy controls, and their response times in error trials were shorter than did controls [18], suggesting difficulties in inhibiting irrelevant stimuli in PIU. Similarly, another study found that poorer performances on the Flanker task predicted more severe symptoms of PIU in college students [19]. Furthermore, higher level of social media use was associated with higher levels of self-report distraction in daily life, and participants with excessive social media use were slower in the color-word Stroop task compared to controls [3]. However, another study did not find a significant effect of social media use on Stroop task performance [20]. How internet-related information interfered with target processing in PIU individuals remains inadequately investigated.

Taken together, whether and how PIU is associated with deficits in prepotent response inhibition and interference control remains unclear and inconclusive. Moreover, although several studies have separately examined the two inhibitory control components among individuals with PIU, there is a lack of research that simultaneously examines both functions to better understand their respective roles in PIU. In addition, among the limited studies that have investigated interference control function in PIU, none of them utilized internet-related stimuli thus limiting the generalizability of these findings to the processing of internet-related information. To address these gaps and achieve a more comprehensive understanding of inhibitory control in PIU, the current study examined prepotent response inhibition and interference control in a group of participants with PIU using the Go/No-Go and the Flanker tasks, with internet-related and internet-unrelated images and words as task stimuli. We hypothesized that participants with PIU would exhibit impaired performance in both the Go/No-Go and the Flanker tasks than controls, particularly in conditions involving internet-related stimuli.

## Method

### Participants

A total of 292 undergraduates were invited to complete the Revised Chinese Internet Addiction Scale (CIAS-R) [21]. The CIAS-R has been commonly used for assessing PIU in Chinese populations, it consists of 26 items on a four-point Likert scale, 1 being ‘does not match my experience’ and 4 indicating ‘definitely matches my experience’, with higher total scores indicating a greater severity of PIU. In line with previous studies [16], individuals who scored higher than 53 on the CIAS-R were categorized as individuals with PIU, and those who scored lower than 46 were classified as controls. Of our participants who completed the CIAS-R-2 (*n* = 292), 37.7% met the inclusion criteria for PIU and 30.1% met the inclusion criteria for controls and were invited to participate in our study. Finally, thirty participants (12 female) who met the PIU criteria and 30 control participants (17 female) were included in the present study. The sample size was determined by a power analysis, with a medium effect size *f* = 0.25, an alpha level of 0.05 and a statistical power of 0.9. All participants had normal color vision and normal or corrected-to-normal visual acuity.

### Experimental design

For the Flanker task, a 2 (Group: PIU vs. Control) × 2 (Stimulus Type: Internet-related words vs. Internet-unrelated words) × 2 (Congruency: Congruent vs. Incongruent) mixed factorial design was used, with Group as a between-subjects variable, and Stimulus type and Congruency as within-subjects variables. For the Go/No-Go task a 2 (Group: PIU vs. Control) × 2 (Stimulus Type: Internet-related vs. Internet-unrelated) mixed factorial design was used, with Group as a between-subjects variable, and Stimulus type as a within-subjects variable. Both tasks were programmed with E-prime 2.0. The stimuli were displayed on a laptop with a 15.6 inch screen, with a screen resolution of 1920 × 1080 and refresh rate of 60 Hz. To control for potential order effects and standardize the testing procedure across participants, the Flanker task was always performed before the Go/No-Go task.

### The Flanker task

To study interference controls in PIU, a variant of the Flanker task was employed, with the internet-related and internet-unrelated words as stimuli.

To ensure that the words used in the study were matched on arousal, valence, and familiarity, and also differed on their relevance to internet, a total of 76 two-Chinese character words were firstly collected. There were 38 internet-related words (e.g., download) and 38 internet-unrelated words (e.g., stairs) respectively. A group of 36 participants were recruited to rate these words on four dimensions: arousal (with 1 being extreme calmness and 7 indicating high arousal), emotional valence (with 1 being extreme unpleasantness and 7 indicating high pleasantness), familiarity (with 1 being extremely unfamiliar and 7 indicating extremely familiar), and relevance to the internet (with 1 being extremely irrelevant and 7 indicating highly relevant). Finally, 40 words were selected, with twenty being internet-related and the remaining 20 being internet-unrelated. The selected internet-related and internet-unrelated words were matched in terms of arousal, emotional valence and familiarity (*ps* > 0.05). As expected, internet-related words (*M* = 6.46, *SD* = 0.83) were rated as significantly more relevant to the internet than internet-unrelated words (*M* = 1.82, *SD* = 1.03), *t* = 27.07, *p* < 0.001.

**Fig. 1:**
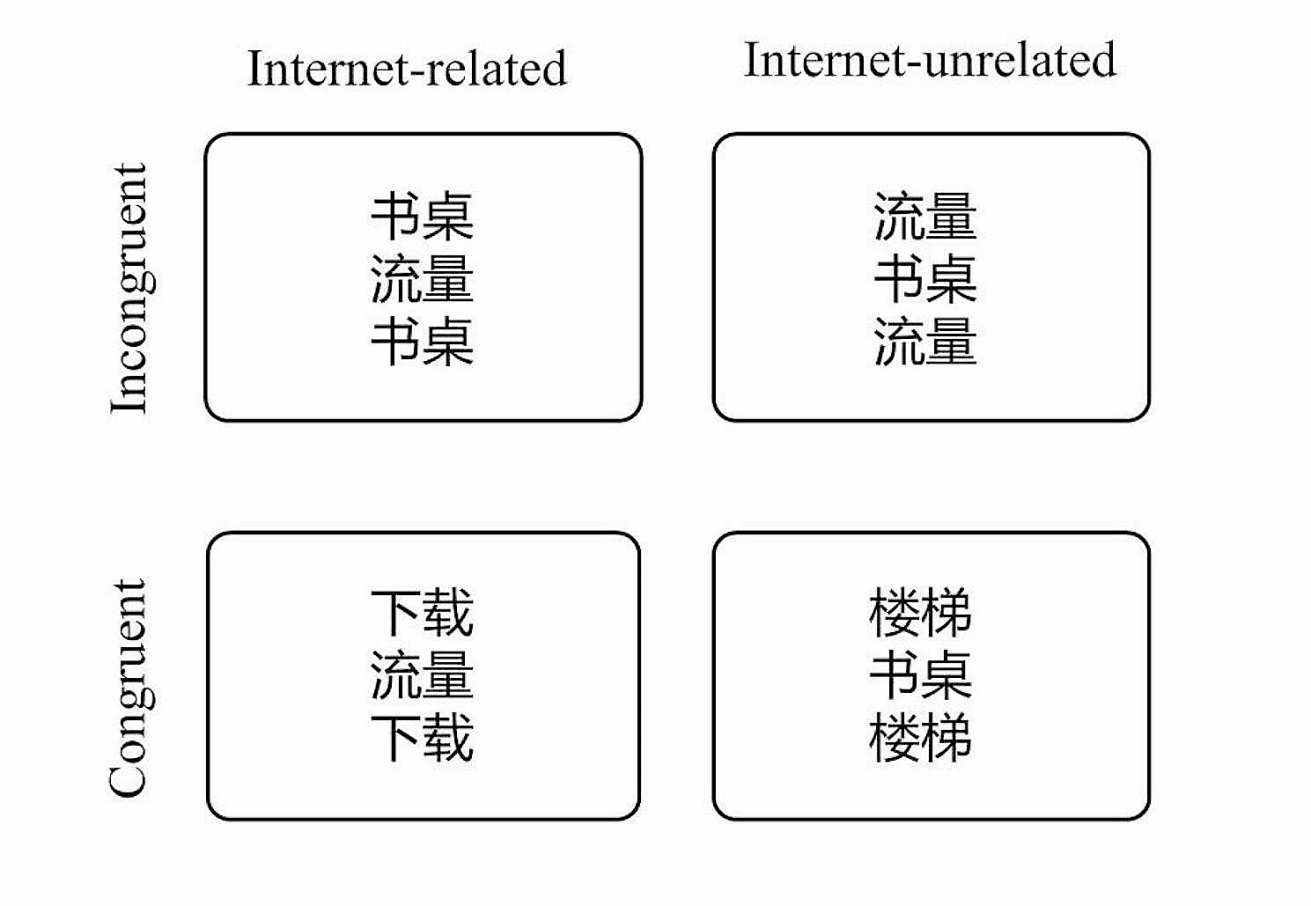
Stimulus display in the Flanker task. Note: 书桌 = desk, 流量 = (mobile) data, 下载 = download, 楼梯 = stairs

In the Flanker task, each trial began with a fixation at the center of the screen for 1000 ms, and one blank screen was then displayed for 20ms-200ms, and then a stimulus array was displayed for 1000 ms, followed by a blank screen for 500ms. Three words were presented, participants were instructed to determine the identity of the word shown (either internet-related or internet-unrelated target) in the middle, while disregarding the Flanker words. The central word and the Flanker words were presented in either a congruent condition, where they belonged to the same category, or an incongruent condition, where they belonged to different categories. The Flanker words were positioned at the upper and lower sides of the central word, as depicted in Fig. 1. Participants were instructed to press “F” for internet-related target and press “J” for internet-unrelated target, as accurately and quickly as possible. After participants provided their response, the stimuli array was immediately removed, and a blank screen was presented for a duration of 500 ms.

The Flanker task consisted of 160 trials, and there were 40 trials for each of the four conditions formed by the combination of Congruency (i.e., congruent, incongruent) and Stimulus type of the target word (i.e., internet-related, internet-unrelated). The sequence of congruent and incongruent trials was pseudorandomized within each block to minimize any potential order effects. Participants were given the opportunity to rest after completing every 50 trials. Prior to the formal experiment, participants underwent a practice block comprising eight trials, with two trials for each condition. The formal experiment would not start until participants achieved an accuracy equal to or higher than 80% during the practice session.

### The Go/No-Go task

To study response inhibition in PIU, the Go/No-Go task was used, with internet-related and internet-unrelated images as stimuli.

To ensure that the images used in the study were matched on arousal and valence, and also differed on relevance to internet, a total of 40 images were firstly collected from the internet. Out of them, 20 images were internet-related (e.g., the WeChat icon) and 20 were internet-unrelated (e.g., an image of a plate). A group of 30 participants who did not participate the formal experiment were recruited to rate these images from three dimensions: arousal (with 1 being extreme calmness and 9 indicating high arousal), emotional valence (with 1 being extreme unpleasantness and 9 indicating high pleasantness), and relevance to the internet (with 1 being extremely irrelevant and 9 indicating highly relevant). Finally, eight images were selected, four of them were internet-related, and four were internet-unrelated. The selected images were matched in terms of arousal and emotional valence (*ps* > 0.05). As expected, internet-related images (*M* = 8.28, *SD* = 0.13) were rated as significantly more relevant to the internet than internet-unrelated images (*M* = 2.23, *SD* = 0.11), *t* = 68.914, *p* < 0.001.

In the Go/No-Go task, each trial began with the display of a fixation at the center of the screen for 500 ms, one image was then displayed for 500ms, followed by a blank screen for 1000ms. Each image was standardized to a resolution of 513 × 384 pixels. Participants were instructed to press “H” for Go trials and withhold response for No-Go trials.

The Go/No-Go task consisted of six blocks, with each block comprising 40 trials. Specifically, three blocks involved internet-related images as the Go stimuli and internet-unrelated images as the No-Go stimuli, while the other three blocks had internet-unrelated images as Go stimuli and internet-related images as No-Go stimuli. Each block consisted 32 Go trials and 8 No-Go trials, which were pseudorandomized. Participants first completed a practice block of eight trials (six of them were Go trials), the formal experiment would not start until participants achieved an accuracy equal to or higher than 80% during the practice session.

### Statistical analysis

Statistical analyses were performed using SPSS 17.0. For the analysis of RTs, trials with incorrect responses or reaction times (RTs) below 200ms were excluded from the analysis.

Group differences between the PIU and control groups in demographic variables were first examined. To investigate group differences on performance of the Go/No-Go task, repeated measures analysis of variances (ANOVAs) were conducted, with Group (PIU group, control group) as the between-subjects factor, Stimulus type (internet-related, internet-unrelated) as the within-subject factor, and the dependent variables were RT in Go trials and the proportion of successful stops (accuracy) in No-Go trials. For the Flanker task, the Group (PIU group, control group) × Congruency (congruent, incongruent) × Stimulus type (internet-related, internet-unrelated) ANOVAs were conducted, the dependent variables were RTs and response accuracy in each condition.

## Results

### Demographic and psychometric variables

Descriptive statistics were listed in Table 1. There was no significant difference on age, years of education, or sex between participants with PIU and controls, *p*s > 0.05. Participants with PIU scored significantly higher on the CIAS-R than controls, *t* (58) = 21.082, *p* < 0.001, *d* = 5.44.

**Table 1:** Demographic variables of participants

	PIU group	Control group	t/χ2	*p*
	(*n* = 30)	(*n* = 30)		
	M ± SD	M ± SD		
Gender (female/male)	17/13	12/18	0.07	0.796
Age (years)	19.80 ± 1.13	19.53 ± 1.55	0.76	0.449
Education (years)	14.08 ± 1.26	13.78 ± 1.26	0.92	0.360
CIAS-R	64.03 ± 5.34	36.73 ± 4.67	21.082	< 0.001

Note. CIAS-R: the Revised Chinese Internet Addiction Scale

### Performance on the Go/No-Go task

Analyses of group differences in RTs of Go trials revealed a significant main effect for Stimulus type, participants responded faster to internet-unrelated stimuli (463 ± 49 ms) than to internet-related stimuli (477 ± 46 ms), *F* (1,58) = 23.43, *p* < 0.001, *η*^2^ = 0.29. Neither the main effect of Group nor the interaction effect between Group and Stimulus type was significant, *F* (1,58) = 0.46, *p* = 0.501, *η*^2^ = 0.008; *F* (1,58) = 2.92, *p* = 0.093, *η*^2^ = 0.048. Descriptive statistics were presented in Table 2.

For accuracy in No-Go trials, the analysis revealed a significant main effect of Stimulus type, participants exhibited poorer performance in inhibiting internet-related stimuli (0.83 ± 0.08) compared to internet-unrelated stimuli (0.91 ± 0.06), *F* (1,58) = 69.55, *p* < 0.001, *η*^*2*^ = 0.55. Neither the main effect of Group nor the interaction effect between Group and Stimulus type was significant, *F* (1,58) = 0.33, *p* = 0.567, *η*^2^ = 0.006; *F* (1,58) = 0.52, *p* = 0.473, *η*^2^ = 0.009. Descriptive statistics were listed in Table 2.

**Table 2:** Task performance on the Go/No-Go and Flanker tasks

Measures	Variables	Stimulus type	PIU group (*n* = 30)	Control group (*n* = 30)^a^
			M ± SD	M ± SD
The Go/No-Go task				
	RTs (Go trials) (ms)	Internet-related	478 ± 47	475 ± 47
		Internet-unrelated	469 ± 53	456 ± 44
	ACC (No-Go trials)	Internet-related	0.83 ± 0.08	0.83 ± 0.08
		Internet-unrelated	0.91 ± 0.07	0.92 ± 0.05
The Flanker task				
	Congruent RTs (ms)	Internet-related	596 ± 36	611 ± 48
		Internet-unrelated	609 ± 46	618 ± 50
	Incongruent RTs (ms)	Internet-related	617 ± 40	634 ± 50
		Internet-unrelated	634 ± 48	625 ± 45
	Congruent ACC	Internet-related	0.93 ± 0.05	0.92 ± 0.08
		Internet-unrelated	0.92 ± 0.07	0.90 ± 0.07
	Incongruent ACC	Internet-related	0.86 ± 0.07	0.85 ± 0.10
		Internet-unrelated	0.86 ± 0.08	0.85 ± 0.08

^a^ One participant from the control group was excluded from the analyses of the Flanker task because the accuracy was lower than 50%.

### Performance on the Flanker task

**Fig. 2:**
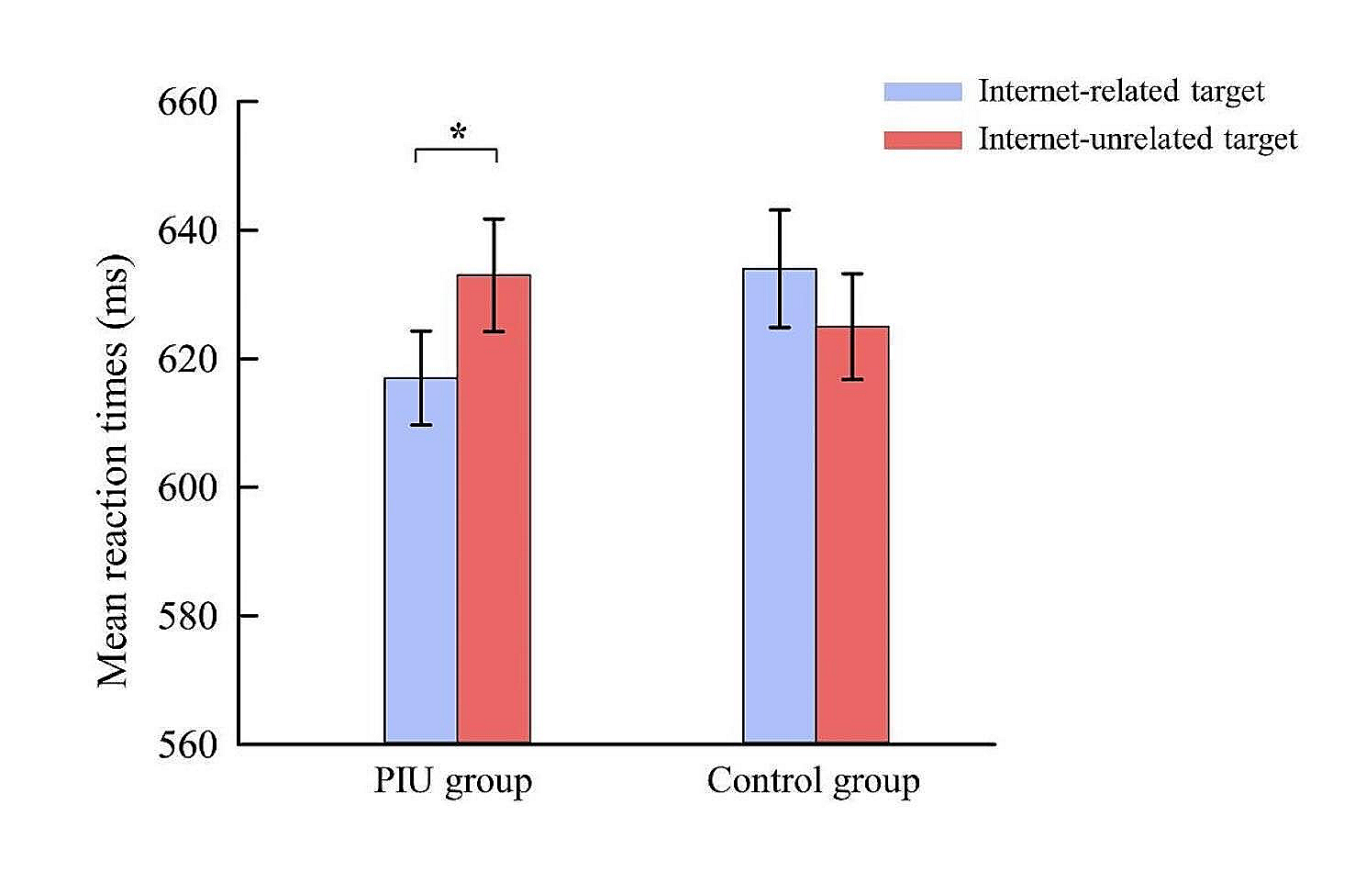
Interaction effect of Group and Stimulus type on RTs in incongruent condition of the Flanker task (error bars represent standard error)

For RTs, the ANOVA revealed a significant main effect of Congruency, longer RTs were observed in incongruent trials compared to congruent trials, *F* (1,57) = 56.74, *p* < 0.001, *η*^2^ = 0.50. The three-way interaction of Group × Stimulus type × Congruency was significant, *F* (1,57) = 4.08, *p* = 0.048, *η*^2^ = 0.07. Further analysis showed a significant interaction effect between Group and Stimulus type in incongruent condition (Fig. 2), *F* (1,57) = 5.58, *p* = 0.022, *η*^2^ = 0.09. Specifically, participants with PIU exhibited longer RTs in conditions with internet-unrelated stimulus as targets (634 ± 48 ms) than in conditions with internet-related stimulus as targets (617 ± 40 ms), *p* = 0.02, that is, internet-related distractors elicited greater interference effect than internet-unrelated distractors in participants with PIU. For the control group, no significant difference on RTs was found between conditions with internet-unrelated targets and conditions with internet-related targets, *p* = 0.882. The interaction effect between Group and Stimulus type in congruent condition was not significant, neither the main effect of Group nor the main effect of Stimulus type was significant, *ps* > 0.05.

For accuracy, the main effect of Congruency was significant, with accuracy in congruent trials (0.92 ± 0.06) being significantly higher than that in incongruent trials (0.86 ± 0.07), *F* (1,57) = 98.76, *p* < 0.001, *η*^2^ = 0.63. No other significant effects were found, *ps* > 0.05.

## Discussion

The current study used the Go/No-Go task and the Flanker task to investigate prepotent response inhibition and interference control function among individuals with PIU. Interestingly, the results showed no significant differences between the PIU and control group in the Go/No-Go task, and all participants responded slower to internet-related Go stimuli but had difficulties withholding the prepotent responses to internet-related No-Go stimuli. However, in the Flanker task, the PIU group showed poorer performance in controlling the interference effect of internet-related information compared to controls. These findings emphasize the varying effects of PIU on the two components of inhibitory control, and highlighting the important role of interference control in relation to PIU.

### Prepotent response inhibition in PIU

Results from the Go/No-Go task showed that individuals with PIU did not differ significantly in prepotent response inhibition from controls. This finding was in line with previous studies [2, 17], which found no difference between participants with PIU and controls on accuracy of No-Go trials. In addition, Chamberlain et al. [15] also found no difference between participants with PIU and controls in withholding the inappropriate prepotent response in the stop-signal task. Nevertheless, we noticed that these findings were not consistent with studies conducted in individuals with IGD [22, 23]. For example, in the study by Yao et al. [23], it was shown that male participants with IGD committed more errors than healthy controls in No-Go trials. This discrepancy might be related to sampling bias, as their study only included male participants due to the higher prevalence of IGD among males. The impact of sex differences on PIU has been documented. A meta-analytic study, which analyzed 115 independent samples from 34 countries, further corroborated that males tend to exhibit a higher tendency towards PIU compared to females [24]. In addition, it is noteworthy that PIU is an umbrella term encompassing various forms of internet overuse problems, whereas IGD represents a distinct subset of PIU characterized by more severe issues specifically related to addiction to internet gaming and has been included as a formal disorder in the 11th revision of the International Classification of Diseases (ICD-11) (ICD-11; WHO, 2019) [25]. Individuals diagnosed with IGD were found to exhibit more pronounced impairments in response inhibition compared to those with overall internet overuse [26].

Furthermore, internet-related stimuli elicited slower responses than internet-unrelated stimuli in Go trials in all participants. Theslower response times to Internet-related stimuli may represent a compensatorymechanism that allows individuals to reduce inhibitory errors. Using thedot-probe task in combination with eye-tracking, longer gaze duration werefound for social networking sites images than for control images [27]. Moreover, all participants were found to demonstrate greater difficulties in inhibiting prepotent responses to internet-related stimuli compared to internet-unrelated stimuli. Internet-related stimuli could hold stronger salience, leading to difficulties in inhibiting internet-related information. However, in the current study, internet-related and internet-unrelated stimuli were matched on emotional valence and arousal, thus differences observed in the study were not due to differences in variations in emotional dimensions between the two types of stimuli. Still, it is premature to draw a definitive conclusion that PIU is not related to impairments in response inhibition, as studies utilizing the stop-signal task revealed poorer response inhibition performances in PIU than controls, and both PIU and controls responded faster to internet-related words than tointernet-unrelated words in Go trials [16]. Future research could benefit from utilizing neurophysiological and neuroimaging techniques to further explore the neural mechanisms involved in the processing of internet-related stimuli among individuals with PIU. Notably, a recent study has reported a larger N2pc for Go trials in individuals with PIU, indicating an early attentional facilitation effect specifically for internet stimuli related to PIU [28].

### Interference control in PIU

In the incongruent condition of the Flanker task, participants with PIU exhibited poorer performance in resisting the distraction from internet-related information than internet-unrelated information, whereas no such effect was found in the control group. This finding is in line with previous study in Flanker task using neutral, internet-unrelated stimuli, which showed that individuals with PIU performed worse than controls [18]. In addition, research has shown that individuals with IGD have difficulties in inhibiting the interference caused by gaming-related contents [29]. Therefore, the current study adds to the existing evidence by suggesting that individuals with PIU specifically struggle with resisting internet-related content, rather than experiencing general impairments in interference control. Under the incongruent condition, characterized by the presentation of two conflicting response options, there is an increased need for conflict monitoring and resolution. Consequently, greater efforts in cognitive control are necessary to effectively control interference from the incongruent flankers. For individuals with PIU, the challenge of disengaging from and suppressing internet-related information, especially when it is unrelated to the current task, may relate to dysfunctions in the prefrontal-parietal network, evidenced by enhanced resting functional connectivity density in the right dorsolateral prefrontal cortex in PIU [30]. Additional research is required to fully understand the causal mechanism linking PIU and the observed changes in brain function.

The divergent performance of individuals with PIU on the Go/No-Go and Flanker tasks adds to the growing body of evidence supporting the dissociation between the two components of inhibitory control. Moreover, neuroimaging studies have provided evidence supporting the unique and distinct nature of prepotent response inhibition and interference control in relation to neural networks. Specifically, prepotent response inhibition relies primarily on the frontal-basal ganglia network, with the right inferior frontal gyrus sending “stop” signals to the primary motor cortex via basal ganglia to inhibit prepotent responses [31]. On the other hand, increased activation during the Flanker task has been observed in the left middle frontal gyrus and left dorsal anterior cingulate [32].

The findings have important implications for the design of cognitive training programs for individuals with PIU, and future research could target enhancing the interference control function in this population. Previous studies conducted in healthy individuals and clinical samples have tested the effectiveness of interference control training in improving the ability to inhibit task-irrelevant information [33, 34], providing support for its efficacy. Importantly, to maximize the training effect for individuals with PIU, it is crucial to include internet-related distractors in the training program.

The present study has some limitations. First, it is important to note that no clinical diagnoses were conducted in our study to determine if participants met the criteria for IGD. As a result, the generalizability of the findings to individuals with IGD is limited. Additionally, PIU encompasses a broad spectrum of behaviors, including online compulsive buying, excessive use of social media platforms, and online gambling. The extent to which these varied forms of PIU share common mechanisms of inhibitory control dysfunction remains to be fully explored. Third, we did not control for word frequency in the flanker task, although we did control for word familiarity. Given that word frequency and word familiarity are two related but distinct psycholinguistic features of language, the potential confounding effects of word frequency in influencing task performance should be controlled for in future research. Furthermore, our experimental tasks followed a fixed order, with the Flanker task administered before the Go/No-Go task. This design choice may introduce order effects that could influence the results. Future studies may benefit from counterbalancing the order of the tasks to mitigate such effects and provide a more robust estimate of inhibitory control across tasks.

## Conclusions

In conclusion, this study examined the prepotent response inhibition and interference control in individuals with PIU. Our findings revealed no significant differences in prepotent response inhibition between individuals with PIU and controls, while they responded slower to Internet-related content in Go trials and made more errors to Internet-related content in No-Go trials. In the Flanker task, compared to controls, individuals with PIU demonstrated significant impairments in controlling the interference effect of Internet-related stimuli. The current study contributes additional evidence supporting the dissociation of the two components of inhibitory control and emphasizes the significant role of interference control in relation to PIU. Further studies are needed to investigate the neural correlates underlying deficits in the control of interference associated with PIU. These findings provide valuable insights for the development of cognitive remediation programs targeting PIU and other forms of addiction.

## Data Availability

The datasets used and/or analyzed during the current study are available from the corresponding author on reasonable request.

## Abbreviations

PIU: Problematic internet use
IGD: Internet Gaming Disorder
RTs: Reaction times
CIAS-R: The Revised Chinese Internet Addiction Scale
